# Zebrafish Models in Therapeutic Research of Cardiac Conduction Disease

**DOI:** 10.3389/fcell.2021.731402

**Published:** 2021-08-04

**Authors:** Rui Gao, Jie Ren

**Affiliations:** Xiamen Cardiovascular Hospital, Xiamen University, Xiamen, China

**Keywords:** cardiac conduction system, cardiac conduction disease, zebrafish, human pluripotent stem cells, differentiation

## Abstract

Malfunction in the cardiac conduction system (CCS) due to congenital anomalies or diseases can cause cardiac conduction disease (CCD), which results in disturbances in cardiac rhythm, leading to syncope and even sudden cardiac death. Insights into development of the CCS components, including pacemaker cardiomyocytes (CMs), atrioventricular node (AVN) and the ventricular conduction system (VCS), can shed light on the pathological and molecular mechanisms underlying CCD, provide approaches for generating human pluripotent stem cell (hPSC)-derived CCS cells, and thus improve therapeutic treatment for such a potentially life-threatening disorder of the heart. However, the cellular and molecular mechanisms controlling CCS development remain elusive. The zebrafish has become a valuable vertebrate model to investigate early development of CCS components because of its unique features such as external fertilization, embryonic optical transparency and the ability to survive even with severe cardiovascular defects during development. In this review, we highlight how the zebrafish has been utilized to dissect the cellular and molecular mechanisms of CCS development, and how the evolutionarily conserved developmental mechanisms discovered in zebrafish could be applied to directing the creation of hPSC-derived CCS cells, therefore providing potential therapeutic strategies that may contribute to better treatment for CCD patients.

## Introduction

Regular heart rhythm, which is essential for efficiently pumping blood throughout the body, relies on the cardiac conduction system (CCS) to initiate and conduct the electrical impulse that controls the rhythmic contractions (Park and Fishman, 2011). The CCS is composed of pacemaker cardiomyocytes (CMs) in the sinoatrial node (SAN), the atrioventricular node (AVN) and the ventricular conduction system (VCS) (van Weerd and Christoffels, 2016). The electrical impulse is initiated from the pacemaker CMs in the SAN, which is located at the junction between the right atrium and the superior caval vein, and then conducted through the atrial CMs and reaches the AVN, where it is delayed by the specialized slow conducting AVN CMs. This delay ensures that the blood is filled in the ventricles before the ventricles contract. Then the impulse is propagated though the fast conducting VCS, which consists of atrioventricular bundle (AVB), or His bundle, the left and right bundle branches (BBs) and the Purkinje fiber network, to activate the ventricular CMs (Mangoni and Nargeot, 2008; Christoffels et al., 2010; Munshi, 2012; van Weerd and Christoffels, 2016).

Defects in development or function of the CCS caused by congenital malformation or diseases can lead to cardiac conduction disease (CCD), which is a potentially life-threatening disorder as arrhythmias induced in CCD may result in insufficient circulation, syncope and even sudden cardiac death (Smits et al., 2005; Wolf and Berul, 2006). Currently the most effective treatment is implantation of an electronic pacemaker, which still has limitations (Rosen et al., 2004; Li, 2012). Thus efforts have been devoted toward developing new treatment strategies for CCD, including trying to generate human pluripotent stem cell (hPSC)-derived or human induced pluripotent stem cell (hiPSC)-derived CCS cells that may be transplanted as biological alternatives. Approaches for such creation of functional CCS cells with high efficiency *in vitro* require detailed knowledge of CCS development *in vivo* since complicated genetic networks regulate the formation of distinctive CCS components in a spatio-temporal-dependent manner. However, the cellular and molecular mechanisms of their development remain to be fully elucidated.

The zebrafish, as a vertebrate animal model, has emerged as a powerful tool to probe these fundamental and crucial questions. Distinct from mouse and chick embryos, zebrafish embryos can survive longer even with the most severe cardiovascular defects by receiving sufficient oxygen through passive diffusion, which permits the phenotypic and functional investigations (Stainier, 2001). Moreover, zebrafish embryos are externally fertilized and optically transparent, which allows live *in vivo* manipulation and imaging during early development (Stainier, 2001). Meanwhile, the zebrafish produces hundreds of offspring and grows very fast, which facilitates identification of mutants perturbing specific genes by utilizing CRISPR-Cas9 technology (Hwang et al., 2013). In particular, even though the zebrafish, as an ectothermic vertebrate organism with low metabolic rate, has only a two-chambered heart (one atrium and one ventricle) with no sophisticated AVN structure and VCS which exist in mammals, it shares well conserved atrial and ventricular structures and possesses conserved pacemaker CMs, slow conducting atrioventricular canal (AVC) CMs, which act as the functional equivalent to the mammalian AVN CMs, and trabecular CMs, which serve as the evolutionary precursors of the Purkinje CMs in mammals (Jensen et al., 2012; van Weerd and Christoffels, 2016). In addition to the functionally conserved cardiac structures and CCS components, the zebrafish also exhibits a high level of genetic conservation with humans (Stainier et al., 1993; Yelon et al., 1999; Chi et al., 2008b), allowing the application of cardiac developmental mechanisms discovered in zebrafish to hPSC or hiPSC system to instruct the creation of hPSC- or hiPSC-derived CCS cells, therefore providing potential therapeutic strategies for CCD. All these features have enabled the zebrafish as a valuable model organism to elucidate the detailed regulatory mechanisms for CCS development and complement CCS studies in other model organisms. In this review, we will highlight not only recent insights into the cellular and molecular mechanisms controlling development of CCS components revealed in zebrafish, but also how these developmental findings could contribute to innovative strategies for generating hPSC- or hiPSC-derived CCS cells that may serve as potential sources for cell therapy or disease modeling, therefore shedding light on the therapeutic treatment for CCD.

### Pacemaker CMs

Pacemaker CMs in the SAN are located at the junction between the right atrium and the superior caval vein in mammals. These specialized CMs spontaneously generate the electrical impulse and maintain the proper heart rhythm (Mangoni and Nargeot, 2008; Christoffels et al., 2010). Although the zebrafish heart is comprised of only two chambers (one atrium and one ventricle), pacemaker CMs have been identified at the junction between the atrium and the sinus venosus through a series of electrophysiologic and molecular studies. Optical mapping analyses using calcium sensitive dye or genetically encoded calcium reporter in zebrafish have revealed that cardiac conduction unidirectionally propagates from the sinus venous to the outflow tract as early as 24 h post fertilization (hpf), indicating the presence of functional pacemaker CMs in the sinoatrial region (Chi et al., 2008b; Panakova et al., 2010). Optogenetic studies combining optical tools and transgenic expression of light-gated ion channels, which can detect regions sensitive to hyperpolarization in the zebrafish heart, have further located pacemaker CMs in the sinoatrial region at 24 hpf (Arrenberg et al., 2010). Moreover, molecular and electrophysiological analyses in zebrafish have characterized CMs at the junction between the atrium and the sinus venosus, which are organized as a ring-shaped structure, as pacemaker CMs since they exhibit typical pacemaker CM action potentials and express similar pacemaker CM molecular markers as identified in mammals, such as Isl1, Shox2, and Hcn4 (Blaschke et al., 2007; Sun et al., 2007; Espinoza-Lewis et al., 2009; Tessadori et al., 2012; Liang et al., 2015; Vedantham et al., 2015). In addition, knocking down these genes leads to bradycardia, a phenotype indicating defects in cardiac pacemaker activity, further supporting their conserved roles for regulating pacemaker development in zebrafish (Blaschke et al., 2007; de Pater et al., 2009; Tessadori et al., 2012). Therefore the existence of molecularly and functionally conserved pacemaker CMs in zebrafish enables thorough investigation into the cellular origins and regulatory mechanisms involved in pacemaker CM development, which may contribute to establishment of potential strategies generating hPSC-derived or hiPSC-derived pacemaker CMs for biological cardiac pacemaker therapy.

**FIGURE 1:**
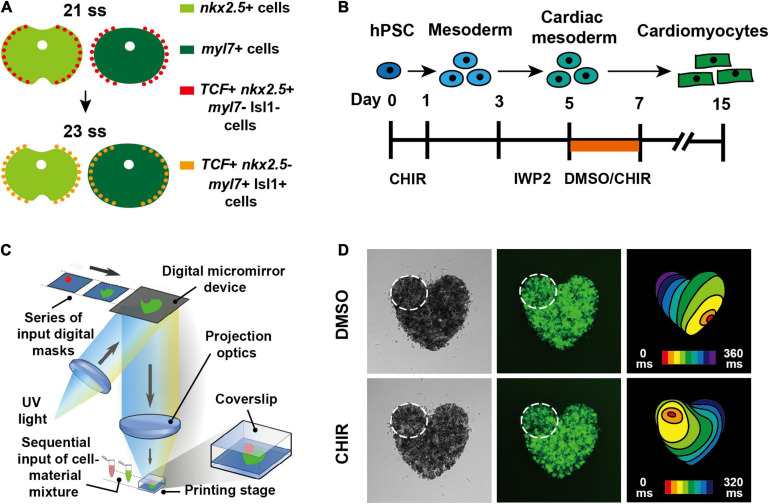
Canonical Wnt signaling promotes pacemaker CM differentiation both in zebrafish and hPSCs (Ren et al., 2019). **(A)** Schematics illustrate that outlying Nkx2.5 + cardiac progenitor cells respond to canonical Wnt signaling and differentiate into pacemaker CMs in zebrafish. **(B)** Schematic illustrates the experimental design for inducing pacemaker-like CMs from hPSCs through activating canonical Wnt signaling. **(C)** Diagram illustrates 3D bioprinting approach. **(D)** hPSC-derived pacemaker-like CMs can pace other CMs in 3D bioprinted mini heart models.

Even though the specialized pacemaker CMs were discovered more than 100 years ago (Keith and Flack, 1907; Trautwein and Uchizono, 1963), their developmental origins remain to be fully defined since they do not retain the expression of the cardiac progenitor marker gene Nkx2.5 after their differentiation, which is distinct from other types of CMs (Wiese et al., 2009). Previous fate mapping studies by means of Dil labeling in mouse embryos have suggested that pacemaker CMs derive from the lateral rim of cardiac mesoderm that down-regulates Nkx2.5 expression prior to their differentiation (Mommersteeg et al., 2010). However, fate mapping analyses by vital dye labeling in chick embryos have reported that pacemaker CMs originate from a tertiary heart field, which is an Nkx2.5 negative region (Bressan et al., 2013). The unclear developmental origins therefore hinder studies searching for the spatio-temporally controlled signaling cues that direct pacemaker CM formation. Compared to mouse embryos, zebrafish embryos are externally fertilized and optically transparent, which are suitable for live *in vivo* manipulation and imaging during early development. In addition, stable transgenic lines labeling specific genes or cell types are easy to establish in zebrafish. Therefore taking advantage of the easy embryonic accessibility and optical transparency during early cardiogenesis, recent studies have utilized a lineage tracing strategy based on the photoconvertible *Tg(nkx2.5:Kaede)* transgenic line in zebrafish and revealed that the outlying Nkx2.5 + progenitors that are located at the most lateral regions of the cardiac mesoderm give rise to pacemaker CMs (Ren et al., 2019). Nkx2.5 + cells at the outlying regions of the cardiac mesoderm are photoconverted at an early developmental stage, and then tracked and identified as Isl1 + /*myl7* + /Nkx2.5- pacemaker CMs at a later stage, supporting previous fate mapping studies in mouse embryos and demonstrating that outlying Nkx2.5 + progenitors are the major developmental source of pacemaker CMs. However, it’s also possible that pacemaker CMs may derive from multiple cardiac progenitor sources given the tertiary heart field studies in chick embryos. Alternatively, the discrepancy may be explained by the developmental stage of the tertiary heart field defined in chick embryos because it is an earlier stage, when the expression domains of cardiac progenitor marker genes may be still changing, than the stages examined in mice or in zebrafish. Meanwhile, canonical Wnt signaling was reported to specify mesodermal cells in the tertiary heart field to the pacemaker lineage in chick embryos (Bressan et al., 2013). Several transcription factors critical for pacemaker CM formation, such as Shox2, Isl1, Tbx18, Tbx3, and Tbx5, have also been identified through a series of mouse studies (Christoffels et al., 2006; Mori et al., 2006; Blaschke et al., 2007; Hoogaars et al., 2007; Sun et al., 2007; Espinoza-Lewis et al., 2009; Wiese et al., 2009; Liang et al., 2015; Vedantham et al., 2015). Although these findings suggest that canonical Wnt signaling may regulate pacemaker CM development, the specific Wnt ligands, the precise mesodermal cells that create pacemaker CMs, and the molecular mechanisms by which canonical Wnt signaling initiates the pacemaker program remain elusive. Based on the cellular origin studies, Wnt5b activated canonical Wnt signaling was discovered in zebrafish to induce the outlying Nkx2.5 + progenitors to differentiate into pacemaker CMs. It has been further reported that canonical Wnt5b signaling directly activates pacemaker differentiation transcription factors Isl1 and Tbx18, and inhibits Nkx2.5 (Figure 1A), demonstrating that it is the key signaling to promote pacemaker CM differentiation (Ren et al., 2019). Whether and how other signals cooperate with canonical Wnt5b signaling to direct pacemaker CM formation in stage- and tissue-dependent manners remain to be elucidated.

Disturbances in human pacemaker CM formation or function due to congenital diseases or aging result in arrhythmias which may require medical intervention (Choudhury et al., 2015). Implantation of electronic pacemakers is currently the most effective treatment, but has drawbacks including the need for battery exchange, device-related infections, a lack of hormonal responsiveness, and the inability to adapt to the heart growth of pediatric patients (Rosen et al., 2004; Li, 2012). Promising alternatives to these electronic devices are biological pacemakers that could be pacemaker CMs derived from hPSCs or hiPSCs, which may be transplanted for cell therapy. Even though CMs have been generated from hPSCs or hiPSCs for regenerative medicine more than a decade ago (Laflamme et al., 2007; Yang et al., 2008; Kattman et al., 2011; Lian et al., 2012; Burridge et al., 2014), the specialized pacemaker CMs derived from hPSCs have not been well developed until recent years due to the lack of sufficient understanding for their developmental mechanisms. By testing signaling pathways that are critical for cardiogenesis, inhibition of both fibroblast growth factor (FGF) and bone morphogenetic protein (BMP) signaling during hPSC differentiation was reported to be able to induce pacemaker-like CM formation (Birket et al., 2015). However, activation of BMP signaling was also found to promote the differentiation of pacemaker-like CMs from hPSCs, which function as a biological pacemaker that is able to pace rat hearts *in vivo* (Protze et al., 2017). In the light of these discrepancies, pacemaker CM differentiation mechanisms revealed from *in vivo* model organisms may help to clarify and instruct the *in vitro* differentiation strategies. Through applying the developmental findings, which have been discovered in zebrafish and have demonstrated that canonical Wnt signaling promotes pacemaker CM formation, to hPSC differentiation, pacemaker-like CMs were successfully generated by activating canonical Wnt signaling during specific differentiation stages (Ren et al., 2019). The pacemaker CMs derived from hPSCs using this strategy not only exhibit typical pacemaker action potentials, but also are able to pace other CMs in 3D bioprinted mini heart models (Figures 1B–D), demonstrating that the pacemaker differentiation mechanisms are evolutionarily conserved between zebrafish and human, thus providing potential strategies for biological pacemaker therapy. Meanwhile, BMP4 expression was found to be up-regulated after activation of canonical Wnt signaling (Ren et al., 2019), suggesting that BMP signaling acts downstream of canonical Wnt signaling and thus supporting that activating BMP signaling is crucial for pacemaker CM differentiation. It was also reported that canonical Wnt signaling promotes pacemaker CM specification of cardiac mesodermal cells derived not only from hPSCs, but also from hiPSCs, supporting that canonical Wnt signaling is critical for pacemaker CM differentiation (Liang et al., 2020). Deeper understanding of coordinated signals controlling pacemaker CM development in zebrafish would further facilitate the increase of the *in vitro* differentiation efficiency, contributing to the development of biological pacemaker therapy.

### AVN CMs

AVN CMs are slow conducting CMs that delay the electrical impulse between the atrium and ventricle so that the blood is filled in the ventricles before the ventricles contract (Bakker et al., 2010). Although the particular AVN structure in mammals is not observed in zebrafish, slow conducting AVC CMs as the functional equivalent to the AVN CMs have been identified at the boundary between the atrium and ventricle in the zebrafish heart as early as 48 hpf. Optical mapping studies using the genetically encoded calcium reporter transgenic zebrafish line have revealed the existence of the AV conduction delay at 48 hpf, indicating the presence of functional AVC CMs at the AV boundary (Chi et al., 2008b). Optogenetic studies detecting regions sensitive to hyperpolarization in the zebrafish heart have also located AVC CMs at the AV boundary at 48 hpf (Arrenberg et al., 2010). Furthermore, electrophysiologic analyses have confirmed their functional AVC CM identities because they exhibit typical AVN CM action potentials similar to those in mammals (Chi et al., 2008b). Meanwhile, detailed cellular examination during AVC CM formation have described their unique conical cell shape, which is distinct from the cell shapes of the neighboring atrial or ventricular CMs (Beis et al., 2005). Moreover, similar molecular marker genes as identified in mammals are expressed in zebrafish AVC CMs, including *tbx2b* and *bmp4* (Harrelson et al., 2004; Ma et al., 2005; Aanhaanen et al., 2011; Verhoeven et al., 2011; Singh et al., 2012). Therefore the existence of functionally and molecularly conserved AVC CMs in zebrafish allows detailed analyses of developmental mechanisms controlling AVC CM formation, which may shed light on potential strategies for creating hPSC- or hiPSC-derived AVN CMs.

**FIGURE 2:**
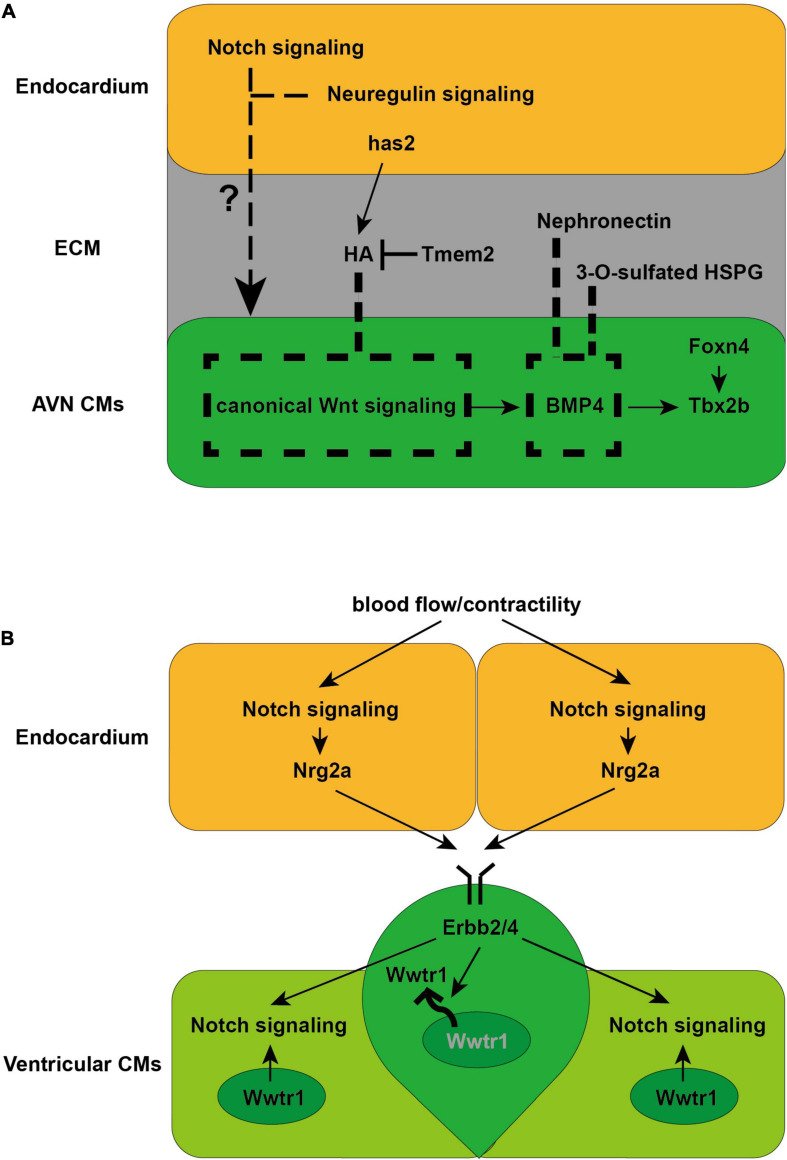
Schematics illustrate the cellular and molecular mechanisms directing AVN and trabeculae CM formation discovered in zebrafish. **(A)** Endocardial Notch and neuregulin signaling are required for AVN CM formation (Milan et al., 2006). Meanwhile canonical Wnt signaling is crucial for AVN CM formation by activating myocardial BMP4 and Tbx2b expression (Verhoeven et al., 2011). ECM-related factors, including HA, Nephronectin and 3-O-sulfated HSPG, limit the distribution of canonical Wnt signaling or BMP4 to confine AVN CM differentiation within the AVC (Patra et al., 2011; Samson et al., 2013; Hernandez et al., 2019). **(B)** Endocardial Notch signaling and Nrg2a is activated by blood flow/contractility, which is received by Erbb2/4 receptor in the CMs to induce trabeculation (Liu et al., 2010; Peshkovsky et al., 2011; Staudt et al., 2014; Samsa et al., 2015; Rasouli and Stainier, 2017). Erbb2 signaling further activates Notch signaling in the neighboring CMs, which acts cell autonomously to inhibits Erbb2 signaling to establish the compact wall architecture necessary for trabeculation (Han et al., 2016). Erbb2 signaling also preserves the architecture of the compact wall by regulating the nuclear localization of Wwtr1, a Hippo pathway effector (Lai et al., 2018).

Previous studies in mice have demonstrated that Tbx2 and Tbx3, which are activated by Bmp2, are crucial regulators for AVN CM development (Harrelson et al., 2004; Hoogaars et al., 2004; Aanhaanen et al., 2011; Singh et al., 2012). Gata4 and Gata6 have also been implicated in AVN CM formation (Munshi et al., 2009; Stefanovic et al., 2014; Stefanovic and Christoffels, 2015). Studies in chick have indicated that Notch signaling in the chamber cardiomyocytes defines the boundary between the AVC and chambers by repressing Bmp2 (Rutenberg et al., 2006), whereas inhibition of Notch signaling in mice results in a hypoplastic AVN and disturbed AV conduction delay (Rentschler et al., 2011), suggesting that AVN CM development requires complicated regulatory networks that act in a spatio-temporal-dependent manner. The zebrafish has offered powerful tools to dissect such complex and detailed cellular events owing to its valuable features including the external fertilization and embryonic optical transparency. Indeed, increasing efforts have been devoted toward understanding how AVC CMs differentiate and establish the AV conduction delay in zebrafish (Figure 2A). These studies have not only illuminated crucial signaling cues that act in a tissue-dependent manner to induce AVC CM formation, but also discovered molecular mechanisms by which AVC CMs are confined within the appropriate cardiac region. Endocardium has been implicated to be required for specification of AVC CMs since *cloche* mutants which lack endocardium fail to develop the AV delay (Milan et al., 2006). Knocking down *notch1b* or *neuregulin* which express in the endocardium leads to failure of AV delay development, suggesting that both endocardial Notch and neuregulin signaling are required for AVC CM formation (Milan et al., 2006). Canonical Wnt signaling has also been reported to regulate AVC CM formation through activating BMP4 and Tbx2b, two critical regulators and marker genes for AVC CMs (Verhoeven et al., 2011). Studies in mice have further confirmed the key role of canonical Wnt signaling since myocardial inhibition of canonical Wnt signaling results in the loss of AVN CMs, whereas ectopic canonical Wnt signaling activation leads to AVN electrical phenotype in the ventricular CMs (Gillers et al., 2015). Transcription factor Foxn4, which regulates Tbx2b expression, has also been shown to be important for AVC formation in zebrafish (Chi et al., 2008a). In addition, a series of studies in zebrafish have revealed that extracellular matrix (ECM), which separates the endocardium and myocardium, and ECM-related factors function to confine AVC CMs to the appropriate location through possibly modulating the presentation of signaling factors to cell surface receptors. ECM protein Nephronectin has been reported to restrain AVC CM formation within the AVC by restricting BMP4 location (Patra et al., 2011). An enzyme 3-O-sulfotransferase-7 (3-OST-7) has also been shown to constrain BMP4 within AVC to confine AVC CM differentiation by modifying glycosaminoglycan (GAG) chains of heparan sulfate proteoglycans (HSPGs), which are cell surface and ECM molecules (Samson et al., 2013). Moreover, hyaluronic acid (HA), another major component of the ECM, which is synthesized by *has2* and degraded by Tmem2, has been revealed to limit the distribution of canonical Wnt signaling and thereby confine the differentiation of AVC CMs within the AVC (Hernandez et al., 2019). Yet, how different signals between the endocardium and myocardium coordinate and direct AVC CM formation remains to be fully elucidated.

Defects in AVN CM formation or function may lead to variable degrees of AV block including complete heart block, which is treated by implantation of electronic pacemakers (Bakker et al., 2010). Alternative therapies need to be developed due to the drawbacks of electronic devices as mentioned before. hPSC- or hiPSC-derived AVN CMs could serve as the possible source for cell therapy or be utilized for *in vitro* disease modeling to improve the treatment for patients. Inhibition of NRG-1β/ErbB signaling has been reported to enhance the formation of hPSC-derived nodal-like cells, which may resemble AVN CMs as similar nodal-like cells can be identified by a chicken GATA6-GFP promoter-enhancer reporter construct that labels the AVN and AVB in the adult mouse heart (Zhu et al., 2010). However, the precise identity of these nodal-like cells requires additional examination and characterization. Therefore strategies for generating AVN CMs from hPSCs or hiPSCs *in vitro* have not been well established yet. Future investigations in zebrafish could further illuminate the cellular origins and differentiation mechanisms of AVN CMs, which would definitely facilitate the development of potential strategies for creation of hPSC- or hiPSC-derived AVN CMs.

### VCS CMs

The VCS is comprised of the AVB, or His bundle, the left and right BBs and the Purkinje fiber network. Distinct from pacemaker CMs and AVN CMs, the VCS CMs are fast-conducting CMs which conduct the electrical impulse rapidly from the AVN to the ventricular working CMs so that the blood is efficiently ejected from the ventricle (Goodyer and Wu, 2018). The AVB and BBs are present in mammals and birds, but not in zebrafish (van Weerd and Christoffels, 2016). Even though the mature Purkinje fiber network may not exist in zebrafish, it is believed that the trabecular CMs in zebrafish are the evolutionary precursors of the Purkinje CMs, because in mammals the Purkinje CMs originate from the trabecular CMs, which serve as the functional equivalent and cellular precursors of the Purkinje fiber network during embryogenesis (Jensen et al., 2012; van Weerd and Christoffels, 2016). Thus investigations for trabecular CM development in zebrafish would provide insight into the complicated regulatory networks controlling the formation of Purkinje CMs, contributing to establishment of novel strategies for generating hPSC-or hiPSC-derived Purkinje CMs.

Previous studies in mice have discovered that endocardial Notch signaling induces ventricular trabecular CM formation by activating Ephrin B2 and Neuregulin1 (Nrg1) (Grego-Bessa et al., 2007; de la Pompa and Epstein, 2012), and ECM remodeling regulated by Notch1 and Nrg1 further promotes the individualization of trabecular units and trabecular rearrangement and growth (del Monte-Nieto et al., 2018). However, the upstream regulators and detailed cellular events and molecular mechanisms controlling this complicated and dynamic developmental process remain to be explored. Utilizing the zebrafish as a unique model organism with embryonic optical accessibility and genetic amenability, a set of profound studies have not only discovered the key role of biomechanical regulation during the trabecular CM formation, but also revealed extensive cellular and molecular mechanisms critical for ventricular trabeculation (Figure 2B). Blood flow/cardiac contractility has been reported to be required for trabeculation in zebrafish, potentially via its role in regulating Notch signaling activation and its downstream Nrg2a expression in the endocardium, which is received by Erbb2/4 receptor in the CMs to induce trabeculation (Liu et al., 2010; Peshkovsky et al., 2011; Staudt et al., 2014; Samsa et al., 2015; Rasouli and Stainier, 2017). These findings are consistent with studies in mice showing that Notch signaling activated endocardial Nrg1 received by ErbB2-4 receptors in the CMs is crucial for trabeculation (Hertig et al., 1999; Grego-Bessa et al., 2007). Interestingly, the reduction of cardiac jelly between ventricular endocardium and myocardium has been suggested to determine the onset of the endocardial-myocardial interactions because the cardiac jelly may constitute a diffusion barrier for Nrg ligands (Rasouli and Stainier, 2017). Detailed examination for cellular events during trabeculation in zebrafish has uncovered that delaminating CMs that form the trabeculae undergo apical restriction and depolarization, along with N-cadherin adhesive junctions relocated from the lateral to the basal side of the CMs, which are processes dependent on Neuregulin signaling and blood flow/cardiac contractility (Cherian et al., 2016; Jimenez-Amilburu et al., 2016). Dynamic localization of Crb2a, a component of the Crumbs polarity complex, has also been reported to be crucial for trabeculation and regulated by blood flow and Nrg/Erbb2 signaling. Crb2a further controls the localization of tight junction protein ZO-1 and adhesive junction protein N-cadherin to ensure proper trabeculation (Jimenez-Amilburu and Stainier, 2019). In particular, myocardial Notch signaling, which is activated by neighboring Erbb2-activated CMs that form the nascent trabeculae, has been discovered to cell autonomously inhibits Erbb2 signaling and prevents CM trabeculation so that the interactive cellular process preserves the architecture of the ventricular myocardial wall in zebrafish (Han et al., 2016). The nuclear localization of Wwtr1, a Hippo pathway effector, has also been reported to be negatively regulated by Erbb2 signaling and critical for establishing the compact wall architecture necessary for trabeculation (Lai et al., 2018). Moreover, Erbb2 signaling has been reported to lead to proliferation-induced cellular crowding which triggers tension heterogeneity among the CMs and drives those with higher contractility to delaminate and form the trabeculae in zebrafish (Priya et al., 2020). Why and how some ventricular CMs can respond to Nrg/Erbb2 signal and become trabeculae remains to be fully elucidated.

Disturbances in the formation or function of the VCS can lead to ventricular arrhythmias, which can result in hemodynamic instability and sudden cardiac death (Scheinman, 2009; Herron et al., 2010; Kim et al., 2014; Iyer et al., 2015; Haissaguerre et al., 2016). hPSCs- or hiPSC-derived Purkinje CMs could be utilized for possible sources of *in vitro* disease modeling or cell therapy. Although the strategy for generating Purkinje CMs from hPSCs or hiPSCs has not been established yet, further detailed investigations of the regulatory networks in zebrafish could provide valuable information toward instructing the creation of hPSCs- or hiPSC-derived Purkinje CMs.

## Conclusion

Disorders in the formation or function of the CCS can lead to potentially life-threatening CCD, which requires implantation of electronic pacemakers. Biological alternatives for distinct CCS components derived from hPSCs or hiPSCs are needed for cell therapy or *in vitro* disease modeling to improve the treatment of patients suffering from CCD due to the drawbacks of the electronic devices. The *in vitro* differentiation strategies to generate such biological alternatives rely on insight into *in vivo* CCS development. The zebrafish has emerged as a valuable animal model organism for dissecting the cellular and molecular mechanisms of CCS development owing to its remarkable features including external fertilization, embryonic optical transparency, a large number of offspring, and embryonic survival ability even without circulation. So far, developmental principles discovered in zebrafish have been successfully applied to hPSC differentiation to promote pacemaker CM formation, not only demonstrating the evolutionarily conserved developmental mechanisms between zebrafish and human, but also providing potential strategies for biological pacemaker therapy (Ren et al., 2019). Even though the cellular and molecular mechanisms underlying the CCS development revealed in zebrafish have started to shed light on the therapeutic studies for human CCD, challenges remain to be addressed in the future. For example, the purity of hPSC- or hiPSC-derived pacemaker CMs still needs to be optimized for further clinical application or disease modeling due to the heterogeneity in the cell population. In addition, the strategies for generating AVN CMs as well as Purkinje CMs from hPSCs or hiPSCs remain to be developed for the potential sources of cell therapy or *in vitro* disease modeling. With the ever-increasing powerful genetic tools, such as single-cell RNA sequencing and single-cell ATAC sequencing which are suitable for heterogeneity analysis (Kumar et al., 2014; Trapnell et al., 2014; Buenrostro et al., 2015; Klein et al., 2015; Farrell et al., 2018; Goodyer et al., 2019; Linscheid et al., 2019; Schiebinger et al., 2019; Liang et al., 2021; Ranzoni et al., 2021), together with well-established CRISPR-Cas9 genomic editing technology and other traditional tools in zebrafish, the cellular and molecular mechanisms controlling CCS development will be further elaborated and provide more insight into the establishment of novel strategies for *in vitro* differentiation of CCS components, thus contributing to the therapeutic studies for human CCD.

## Author Contributions

RG and JR wrote the manuscript. Both authors made substantial contributions to the conception of this reviewed and approved the submitted version.

## Conflict of Interest

The authors declare that the research was conducted in the absence of any commercial or financial relationships that could be construed as a potential conflict of interest.

## Publisher’s Note

All claims expressed in this article are solely those of the authors and do not necessarily represent those of their affiliated organizations, or those of the publisher, the editors and the reviewers. Any product that may be evaluated in this article, or claim that may be made by its manufacturer, is not guaranteed or endorsed by the publisher.
